# Synthesis of Natural-Product-Like Molecules with Over Eighty Distinct Scaffolds**

**DOI:** 10.1002/anie.200804486

**Published:** 2008-11-17

**Authors:** Daniel Morton, Stuart Leach, Christopher Cordier, Stuart Warriner, Adam Nelson

**Affiliations:** * School of Chemistry and Astbury Centre for Structural Molecular Biology, University of LeedsLeeds, LS2 9JT (UK) Fax: (+44) 113-343-6565 E-mail: a.s.nelson@leeds.ac.uk Homepage: http://www.asn.leeds.ac.uk

**Keywords:** cascade chemistry, diversity-oriented synthesis, metathesis, molecular scaffolds, natural products


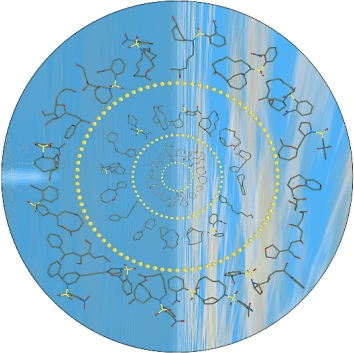


A key challenge in chemical biology is the design and synthesis of compound libraries spanning large tracts of biologically relevant chemical space.[1]–[3] Libraries with high molecular shape diversity, a prerequisite for broad biological activity,[2] are particularly valuable in phenotypic screens. Despite this, organic chemistry is dominated by a remarkably small number of molecular scaffolds: in a recent study[4] of known cyclic organic molecules, 0.25% of the molecular frameworks were found in 50% of the known compounds! The uneven distribution of frameworks may reflect the way that chemical space is generally explored, with chemists tending to prepare compounds based on frameworks that are already known.[4]

Diversity-oriented synthesis (DOS) involves the preparation of compound libraries with high substitutional, stereochemical, and/or scaffold diversity.[5] Varying molecular scaffolds is challenging though ingenious innovations have allowed the parallel synthesis of libraries based on multiple (ca. 6–30) scaffolds.[6] Developing approaches in which the ethos of DOS is retained—that is, that the synthesis is deliberate and simultaneous—is, nonetheless, extremely demanding. High-throughput screens of DOS libraries have, however, yielded useful small molecule tools for chemical genetic studies of cellular protein functions.[7]

Our approach to the combinatorial variation of molecular scaffolds involved the attachment of pairs of unsaturated building blocks to a fluorous-tagged linker (Scheme 1).[8] The fluorous tag allowed the removal of excess reagents at each stage by fluorous-solid phase extraction (F-SPE) alone.[9] We used two general types of building blocks: “propagating” and “capping” building blocks (Scheme 2). To increase the structural diversity of the final compounds, we attached the building blocks using combinations of temporary silaketal tethers[10] and bonds that would remain as a vestige in the final compounds. For example, the cyclopentene building block **9** could be attached to the linker (**1** or **2**) using either a Fukuyama–Mitsunobu reaction[11] (→**3**) or a diisopropylsilylene tether (→**4**). Following deacetylation, a “capping” building block, such as **15**(Sil,All)→**5** or **25**→**6**, was attached to yield metathesis substrates. Metathesis cascades were used to “reprogram” the scaffolds of the metathesis substrates to yield those of the final products (for example **5**→**7** or **6**→**8**). Each metathesis cascade was expected to initiate at the terminal alkene[12] of the “capping” building block, leading to the release of only cyclized products from the fluorous-tagged linker. It was expected that the ability to vary the pairs of building blocks used, together with the nature of the attachment reactions, would yield a small molecule library with extremely high scaffold diversity.

**Scheme 1:**
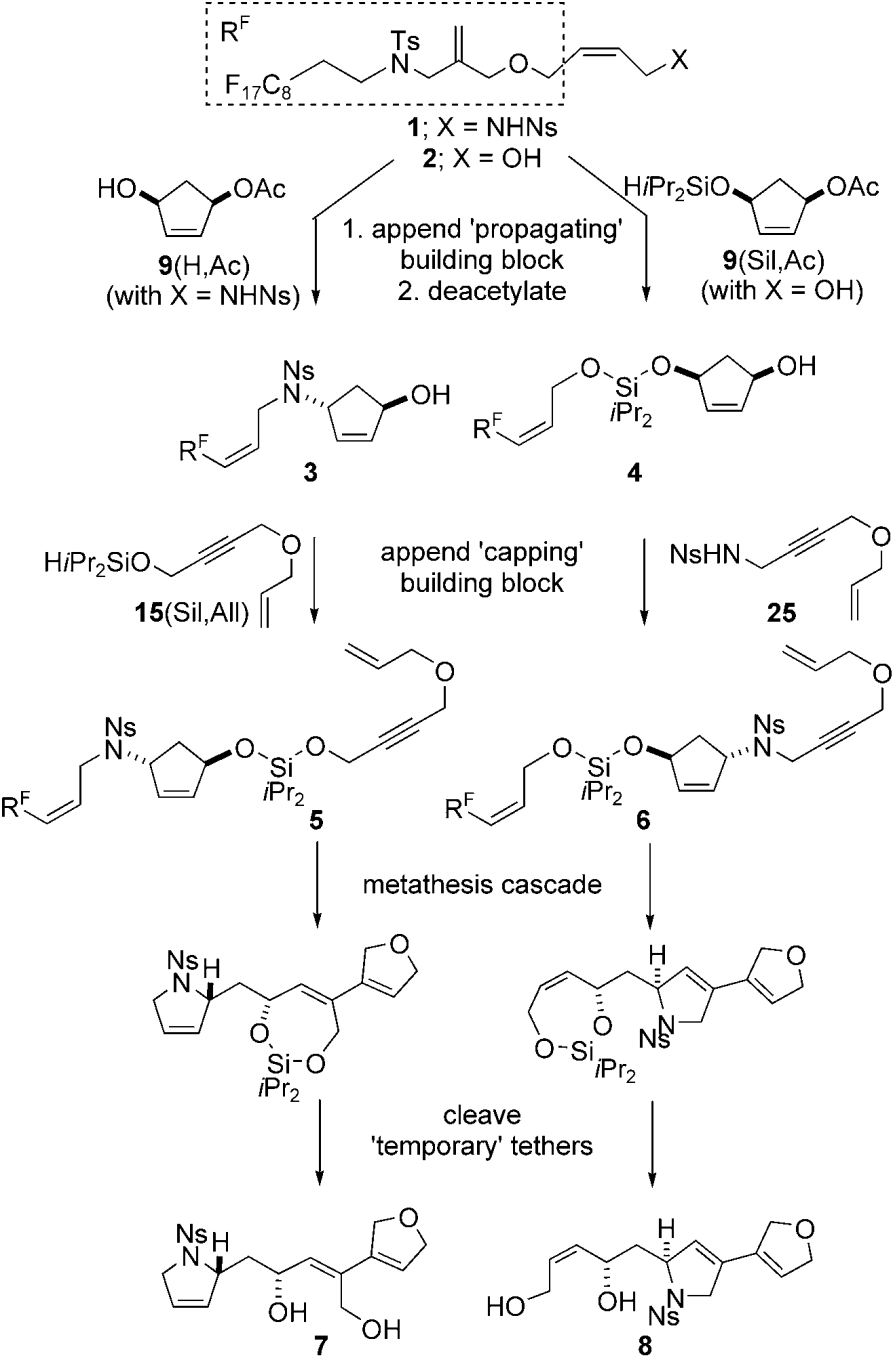
Outline of our approach to the combinatorial variation of the scaffolds of small molecules. The labels in parentheses define the substituents: Ac=acetyl; Sil=diisopropylsilyl; All=allyl.

**Scheme 2:**
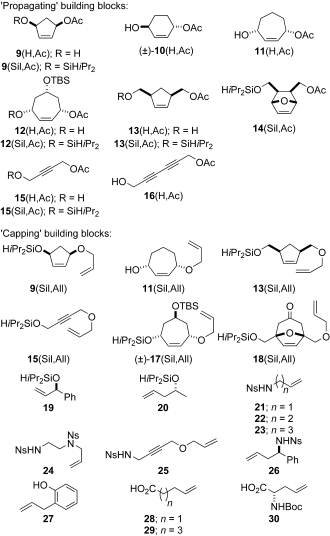
Structures of the building blocks used in our diversity-oriented approach. Enantiomeric structures are distinguished thus: **9** and **9**′.

The building blocks were prepared on a multi-gram scale. Chiral building blocks were generally prepared in enantiomerically-enriched form, often using enzymatic desymmetrisation[13] to induce asymmetry. We started by assessing the proficiency of some propagating building blocks in simple metathesis cascades (see Supporting Information). Diallylated derivatives of the building blocks **9**, **12**, **13**, **14**, **17**, and **18** were metathesized, and a range of products with alternative scaffolds were obtained. The most promising results were fed into the design of our synthetic scheme.

The propagating building blocks were first attached to the fluorous-tagged linker, with, in general, removal of excess reagents by F-SPE alone (Table 1). Thus, the building blocks **9**(H,Ac), **9**′(H,Ac), (±)-**10**(H,Ac), **11**(H,Ac), **12**(H,Ac), **13**(H,Ac), **15**(H,Ac), and **16**(H,Ac) were attached in good to excellent yield to the fluorous-tagged sulfonamide **1** using a Fukuyama–Mitsunobu reaction.[8] In addition, the building blocks **9**(Sil,Ac), **12**(Sil,Ac), **13**(Sil,Ac), **14**(Sil,Ac), and **15**(Sil,Ac) were activated and attached[14] via a diisopropylsilylene tether to the fluorous-tagged alcohol **2**. Some of the silaketal formations did not proceed to completion: filtration through a short pad of florisil allowed the unreacted fluorous-tagged alcohol **2** to be removed. Each intermediate was deacetylated in excellent yield by treatment with saturated ammonia in methanol and the products were used without purification (Table 1).

**Table 1:** Attachment of propagating building blocks and deacetylation.

Attachment of propagating building block			Deacetylation	
Linker	Building block[a]	Mass recovery[b] (purity[c])	Product[d]	Mass recovery[b] (purity[c])
**1**	**9**(H,Ac)	>98% (98%)	**3**	>98% (95%)
**2**	**9**(Sil,Ac)	85%[e] (>95%)	**4**	>98% (93%)
**1**	**9′**(H,Ac)	>98% (98%)	**3′**	>98% (95%)
**1**	(±)-**10**(H,Ac)	>98% (90%)	(±)-**31**	>98% (90%)
**1**	**11**(H,Ac)	>98% (90%)	**32**	>98% (90%)
**1**	**12**(H,Ac)	>98% (90%)	**33**	>98% (90%)
**2**	**12**(Sil,Ac)	88%[e] (>95%)	**34**	>98% (95%)
**1**	**13**(H,Ac)	>98% (89%)	**35**	>98% (90%)
**2**	**13**(Sil,Ac)	61% (>95%)	**36**	>98% (86%)
**2**	**14**(Sil,Ac)	58%[e] (>95%)	**37**	>98% (94%)
**1**	**15**(H,Ac)	>98% (>95%)	**38**	>98% (>95%)
**2**	**15**(Sil,Ac)	95%[e] (90%)	**39**	>98% (90%)
**1**	**16**(H,Ac)	96% (>95%)	**40**	>98% (90%)

[a] Method for attachment to **1**: 1) building block (4 equiv), DEAD (4 equiv), PPh_3_ (4 equiv), THF, 0°C, 1 h; 2) F-SPE; Method for attachment to **2**: 1) building block (5.5 equiv), NBS (5 equiv), CH_2_Cl_2_, 0°C→RT, 15 min; 2) inverse addition of fluorous-tagged linker, DMAP (50 mol%), Et_3_N (15 equiv), 0°C→RT; 3) F-SPE;

[b] Purification by F-SPE only unless otherwise indicated.

[c] Determined by analytical HPLC or 500 MHz ^1^H NMR spectroscopy.

[d] Method: saturated NH_3_ in MeOH.

[e] Purified, in addition, by filtration through a pad of florisil.

Next, “capping” building blocks were attached through silaketal formation, esterification or a variant of the Mitsunobu reaction. In general, F-SPE alone was used to purify each of the resulting metathesis substrates. In total, 86 metathesis substrates were prepared; a wide range of selected examples are shown in Scheme 3.

**Scheme 3:**
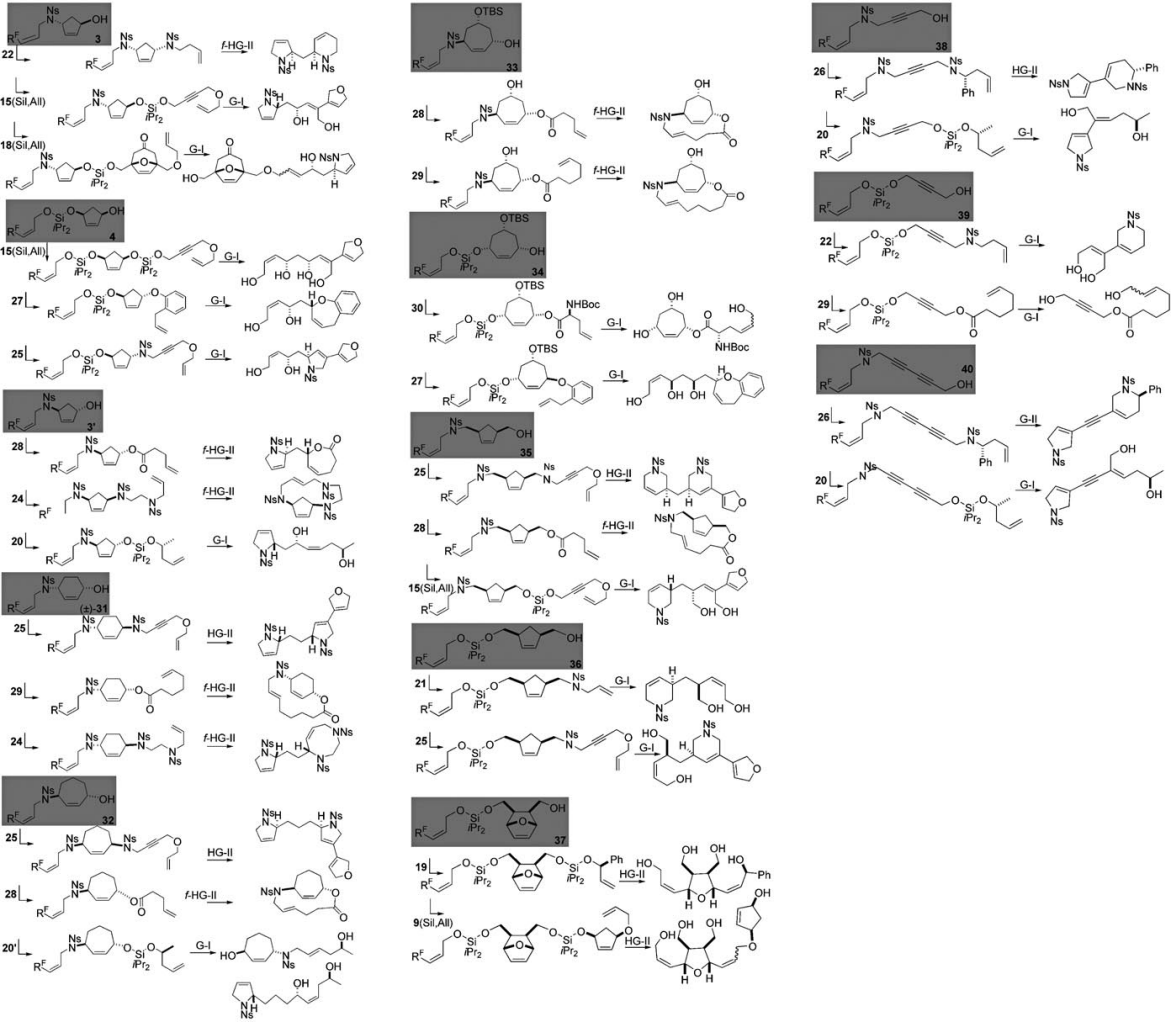
Selected examples of metathesis cascades leading to skeletally diverse products. Methods: Attachment of **21**–**26**: 1) building block (4 equiv), DEAD (4 equiv), PPh_3_ (4 equiv), THF, 0°C, 1 h; 2) F-SPE. Attachment of **27**: 1) building block (4 equiv), DEAD (4 equiv), PPh_3_ (4 equiv), THF, 0°C → reflux; 2) F-SPE. Silaketal formation: 1) building block (5.5 equiv), NBS (5 equiv), CH_2_Cl_2_, 0°C → RT, 15 min; 2) inverse addition of substrate (1 equiv, 0.2m), DMAP (50 mol%), Et_3_N (15 equiv), 0°C → RT; 3) F-SPE. Attachment of **28**–**30**: 1) building block (2 equiv), EDC (2 equiv), DMAP (10 mol%), CH_2_Cl_2_, RT; 2) F-SPE. G-I: 1) catalyst **G** **I**, CH_2_Cl_2_, 45°C; 2) Et_3_N (86 equiv), P(CH_2_OH)_3_ (86 equiv), then silica, then filter through Celite; 3) F-SPE; 4) HF⋅pyridine, THF, then Me_3_SiOMe. HG-II: 1) catalyst **HG** **II**, CH_2_Cl_2_, 45°C; 2) Et_3_N (86 equiv), P(CH_2_OH)_3_ (86 equiv), then silica, then filter through Celite; 3) F-SPE. *f*-HG-II: 1) catalyst ***f-*****HG** **II**, CH_2_Cl_2_, 45°C; 2) F-SPE. DEAD=diethyl azodicarboxylate; NBS=*N*-bromosuccinimide; DMAP=4-dimethylaminopyridine; EDC=*N′*-(3-dimethylaminopropyl)-*N*-ethylcarbodiimide.

The metathesis substrates were each treated with a metathesis catalyst in refluxing dichloromethane (see Scheme 3 for selected examples, and Supporting Information). In general, **G I** (Scheme 4) was used with substrates in which one or both of the building blocks had been attached using a silaketal tether;[14] with other substrates, the fluorous-tagged catalyst[15] ***f-*****HG II** was usually used. The reactions were monitored, and additional catalyst was added as required. The phosphine, P(CH_2_OH)_3_,[16] was used to remove the catalyst **G I**. F-SPE was used to separate the released metathesis products from possible fluorous-tagged contaminants: remaining substrate, the remnant of the linker,[8] unreleased by-products, and, for reactions catalysed by ***f-*****HG II**, the catalyst. The products derived from silaketal substrates were treated with HF⋅pyridine and quenched with Me_3_SiOMe. Finally, the released products were purified.

**Scheme 4:**
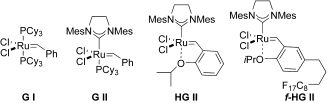
Some catalysts for metathesis reactions.

The linkers **1** and **2** were instrumental to the success of our approach.[8] First, the fluorous tag allowed the assembly of the metathesis substrates without column chromatography. Second, linker design ensured that only metathesized products were released. Thus although yields varied widely, F-SPE enabled the very easy isolation of just the released metathesis products which were subsequently purified. The average yield of the released products, based on the purity of the metathesis substrates, was 46%.

Our synthetic approach yielded products with a diverse range of scaffolds. In most cases, all of the unsaturated groups in the substrate were involved in the dominant metathesis pathway (for example, **41**→**42**; Scheme 5). However, competition between the formation of different ring sizes extended the structural diversity possible. For example, the alkene in the propagating building block of **43** did not participate in the subsequent cascade, and thus a bridged macrocycle (**44**) was formed. In four cases, the competition between alternative cyclizations was sufficiently close that products with two distinct scaffolds were isolated from the same reaction. The metathesis substrate **45** has two more methylene groups than **41**, which had a key effect on its fate: competition between seven- and thirteen-membered ring formation was close, and, after desilylation, two products (**46** and **47**) were obtained.

**Scheme 5:**
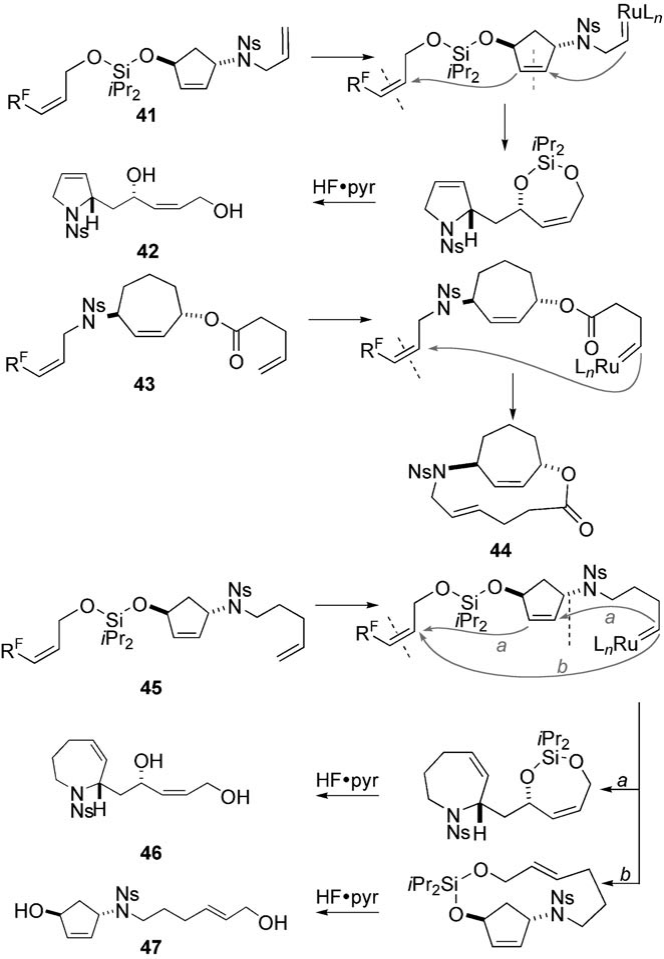
Fate of some metathesis cascade reactions.

The diversity of organic compounds has often been analysed in a chemically intuitive manner in terms of the ring systems in structures.[2],[14],[18] We have assessed the skeletal diversity of our library in terms of an hierarchical approach that formalises the relationship between molecular scaffolds (Figure 1).[17] In this approach, terminal side chains are pruned to give a scaffold which is defined by the rings and unsaturated groups that rigidify the molecule. Rings are then iteratively removed according to intuitive prioritisation rules to reveal the last remaining ring—the “parental” scaffold. The “parental” scaffold is related to scaffolds at lower levels of hierarchy until the molecular scaffold is ultimately reached.

**Figure 1:**
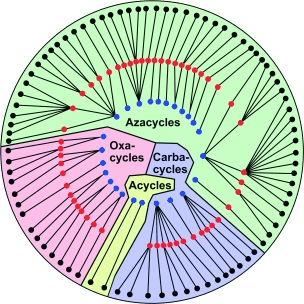
Hierarchical classification which illustrates the relationship between parental scaffolds (blue), daughter scaffolds (red), and molecular scaffolds (black).

The vast majority of the 96 products prepared have a distinct molecular scaffold. However, the scaffold diversity of the library is even more striking at higher levels of the hierarchy. This scaffold tree, like that of natural products,[3] is dominated by the three classes of cyclic compounds: azacycles, oxacycles, and carbacycles. There are 25 parental scaffolds in total, and all ring sizes from 5 to 15 (except 10) are represented at this level. The parental scaffolds are related to 54 “daughter” scaffolds, which are ultimately related to the 84 molecular scaffolds in the library. At all levels of hierarchy, the scaffolds represented had unprecedented diversity. Furthermore, around 65% of the deprotected scaffolds are not found in molecules that have been previously prepared.

Our synthetic approach allowed the systematic exploration of chemical space defined by scores of different molecular scaffolds. Each final compound was prepared from the building blocks in either four or five steps. Column chromatography was avoided at all intermediate stages, facilitating the application of the approach in parallel format. Remarkably, our approach used combinations of just six reaction types: the Mitsunobu reaction, silaketal formation, esterification, deacetylation, metathesis, and desilylation. The ethos of DOS was firmly retained since compounds with >80 scaffolds were prepared using a small number of optimised procedures.

Excitingly, the compounds prepared had many of the broad structural features that are reminiscent of, and have evolved to constrain, natural products: isolated, fused, and spirocyclic ring systems, intramolecular hydrogen bonding motifs, unsaturation, and dense substitution. In this sense, many of the compounds prepared are natural product-like. As with polyketide biosynthesis,[19] a wholly remarkable number of scaffolds with these broad structural features was accessible using, in combination, a relatively small repertoire of fundamental reaction types.

The key to our approach was the extraordinary scope of ring-closing metathesis,[20] which allowed the preparation of a library of much higher skeletal diversity than previous DOS approaches. Combinatorial variation of molecular scaffold was possible through the assembly of alternative metathesis substrates from pairs of simple building blocks. The range of accessible scaffolds was further extended both by varying the linkages between building blocks, and competition between the formation of alternative rings. Many of the diverse scaffolds prepared have scope for easy further diversification which may allow the discovery of novel bioactive small molecule tools.

## Experimental Section

Experimental procedures, characterisation data, and NMR spectra for all novel compounds are provided in the Supporting Information.
